# Screening for Refractive Errors Among Nursing Students Using the WHOeyes Application in Shivamogga City

**DOI:** 10.7759/cureus.97387

**Published:** 2025-11-20

**Authors:** Anupama K, Balu PS, Gayatri Mahadev, Vanamala Satish, Shwetha TM, Swathi H J

**Affiliations:** 1 Community Medicine, Subbaiah Institute of Medical Sciences, Shivamogga, IND; 2 Centre for AI Research and E-Health, Subbaiah Research Institute, Shivamogga, IND; 3 Ophthalmology, Subbaiah Institute of Medical Sciences, Shivamogga, IND; 4 Nursing, Subbaiah Institute of Nursing, Shivamogga, IND

**Keywords:** nursing, refractive error, screening, students, whoeyes

## Abstract

Background: Refractive errors are one of the most frequent causes of visual impairment globally and remain largely preventable with early screening and correction. Many people suffer from uncorrected refractive errors, even though the interventions are effective and low-cost. Healthcare students, especially nursing students, are at high risk because they spend a long time doing near work and watching screens, but vision screening is not widely practiced in most institutions. This study aims to assess the prevalence of refractive errors among nursing students in Shivamogga city based on the WHOeyes application (World Health Organization, Geneva, Switzerland) and to determine the risk factors associated with refractive errors.

Materials and methods: This cross-sectional study was conducted among nursing students of Subbaiah Institute of Nursing, Shivamogga, from January to June 2025. Screening of 577 students aged ≥18 years was done with the WHOeyes app. Distance vision was measured at 2 meters, and classification of visual impairment was mild (visual acuity (VA) <6/12-6/18), moderate (VA <6/18-6/60), severe (VA <6/60-3/60), or blindness (VA <3/60). Socio-demographic information was noted. Data were processed employing descriptive statistics, chi-square test, and other relevant tests of significance, with p < 0.05 being significant.

Results: Out of 577 participants, most were 20-22 years old (52.3%), females (84.7%), and rural residents (58.4%). The largest group of students represented socioeconomic class III (36.4%). In the right eye, most participants had normal vision (87.7%), followed by mild (7.6%), moderate (4.2%), and severe impairment (0.5%). In the left eye, normal vision was seen in 84.1%, with mild (12.0%), moderate (2.8%), and severe impairment (1.2%).

Conclusion: This study emphasizes the role of regular vision screening among nursing students to detect refractive errors at an early stage, referral at the appropriate time, and increased awareness about ocular health. Incorporation of such screening programs in schools can facilitate a decrease in the load of uncorrected refractive errors and ensure eye health among future healthcare professionals.

## Introduction

Refractive errors are among the most common causes of visual impairment globally, but they are preventable with early diagnosis and uncomplicated corrective measures such as spectacles. According to the World Health Organization (WHO), approximately a hundred million people have visual impairment caused by uncorrected refractive errors, despite the availability of effective and affordable remedies [1]. Myopia, hyperopia, and astigmatism constitute the majority of these refractive errors, and their prevalence has been increasing, particularly among adolescents and young adults due to lifestyle changes and prolonged near work [2].

Healthcare students, especially those in nursing, require optimal visual function for academic performance and effective clinical practice. However, due to long study hours and screen exposure, they may be at increased risk of developing or worsening refractive errors [3]. Despite being part of the healthcare system, many nursing students may remain unaware of their own ocular health status, and vision screening is not routinely practiced in many institutions [4].

The WHO has developed a simplified eye screening tool that can be used at the community level or in primary healthcare settings to identify individuals with potential visual impairment and refer them for appropriate care [5]. Utilizing these standardized guidelines can facilitate early detection, reduce the burden of uncorrected refractive errors, and promote eye health awareness, especially among future healthcare providers.

In the context of India, where the burden of uncorrected refractive errors remains high, there is limited data on vision status among nursing students. This study aims to assess the prevalence of refractive errors among nursing students in Shivamogga city using the WHO eye screening guidelines, with the goal of early identification and appropriate referral for vision correction. The study also aims to assess the prevalence of refractive errors among nursing students in Shivamogga city using the WHOeyes application (World Health Organization, Geneva, Switzerland) and to determine factors associated with refractive error.

## Materials and methods

Study setting and duration

The research was carried out at the Subbaiah Institute of Nursing, Shivamogga, over a six-month study period (January to June 2025).

Study design

This was a cross-sectional study designed to assess the prevalence of visual impairment among nursing students using a smartphone-based application and to evaluate associated risk factors.

Study population

All nursing students aged 18 years and above enrolled at the institution were considered eligible.

Sampling technique

A universal sampling technique was applied. Every student who met the eligibility criteria and consented to participate was included in the study.

Inclusion and exclusion criteria

Inclusion Criteria

Individuals aged above 18 years and those who were willing to give consent were included in the study.

Exclusion Criteria

Students wearing spectacles and those with other types of visual impairment were excluded from the study.

Materials and methods

After obtaining clearance from the Institutional Ethical Committee, the study was conducted among nursing college students from January to June 2025. All nursing students were screened for refractive errors using the WHOeyes app (iOS version). The WHOeyes is a validated tool developed by the WHO.

Each student was asked to undergo vision testing for both eyes using the application. During the procedure, participants were asked to stand at a distance of 2 meters from the device. One eye was covered at a time, and the other was tested. Students were instructed beforehand to identify the orientation of the “E” optotypes displayed on the screen by indicating the direction of the arms of the letter. The test was continued until completion, after which the application generated the results.

The WHOeyes app (iOS) incorporates built-in automated distance calculation technology, which ensures accurate measurement of the required distance (2 meters) between the screen and the participant during testing.

The application provides a score (0-100%) along with a fraction corresponding to the participant’s visual acuity. Based on this, vision impairment was diagnosed and classified according to the WHO definitions as follows: mild impairment: visual acuity worse than 6/12 to 6/18; moderate impairment: visual acuity worse than 6/18 to 6/60; severe impairment: visual acuity worse than 6/60 to 3/60; blindness: visual acuity worse than 3/60.

Statistical analysis

Quantitative data were expressed as mean ± SD. Qualitative data were expressed as numbers and percentages. Chi-square tests and other suitable tests of significance were applied at the time of statistical analysis. P-values of <0.05 were considered significant.

## Results

Table 1 shows the socio-demographic details of the study participants. Out of 577 study participants, the majority of them belonged to the age group of 20-22 years (302, 52.33%), followed by <20 years (138, 23.91%), 22-24 years (124, 21.49%), and >24 years (13, 2.2%). Out of 577 study participants, the majority were females (489, 84.74%), and the remainder were males (88, 15.25%). The majority of them belonged to class 3 (210, 36.39%), followed by class 2 (198, 34.31%). The majority of them belonged to rural areas (330, 57.19%), and the remaining were from urban areas (247, 42.81%). The majority of them presented with headache (89, 15.42%), followed by blurring of vision (52, 9.01%) and watering eyes (28, 4.85%). The majority of them were found to have screen time between two and four hours (328, 56.84%), followed by less than two hours (182, 31.54%) and more than four hours (67, 11.62%), respectively.

**Table 1:** Socio-demographic details of the study participants.

Socio-demographic details	Sub-variables	Frequency (n)	Percentage (%)
Age	<20 years	138	23.91%
	20-22 years	302	52.33%
	22-24 years	124	21.49%
	>24 years	13	2.2%
Gender	Male	88	15.25%
	Female	489	84.74%
Socio-economic status	Class 1	46	7.97%
	Class 2	198	34.31%
	Class 3	210	36.39%
	Class 4	123	21.31%
Place of residence	Urban	247	42.81%
	Rural	330	57.19%
Complaints	Headache	89	15.42%
	Blurring of vision	52	9.01%
	Watering of eyes	28	4.85%
	Nil	408	70.7%
Screen time	<2 hours	182	31.54%
	2-4 hours	328	56.84%
	>4 hours	67	11.62%
Total		577	100%

Table 2 shows the visual impairment among study participants. The majority of participants had normal vision in the right eye (506, 87.69%), followed by mild vision impairment (44, 7.62%), moderate vision impairment (24, 4.15%), and severe vision impairment (3, 0.51%).

**Table 2:** Visual impairment among study participants.

Variables	Visual impairment	Frequency	Percentage
Right eye vision	Normal	506	87.69%
	Mild	44	7.62%
	Moderate	24	4.15%
	Severe	3	0.51%
Left eye vision	Normal	485	84.05%
	Mild	69	11.95%
	Moderate	16	2.77%
	Severe	7	1.21%
Total		577	100%

**Figure 1:**
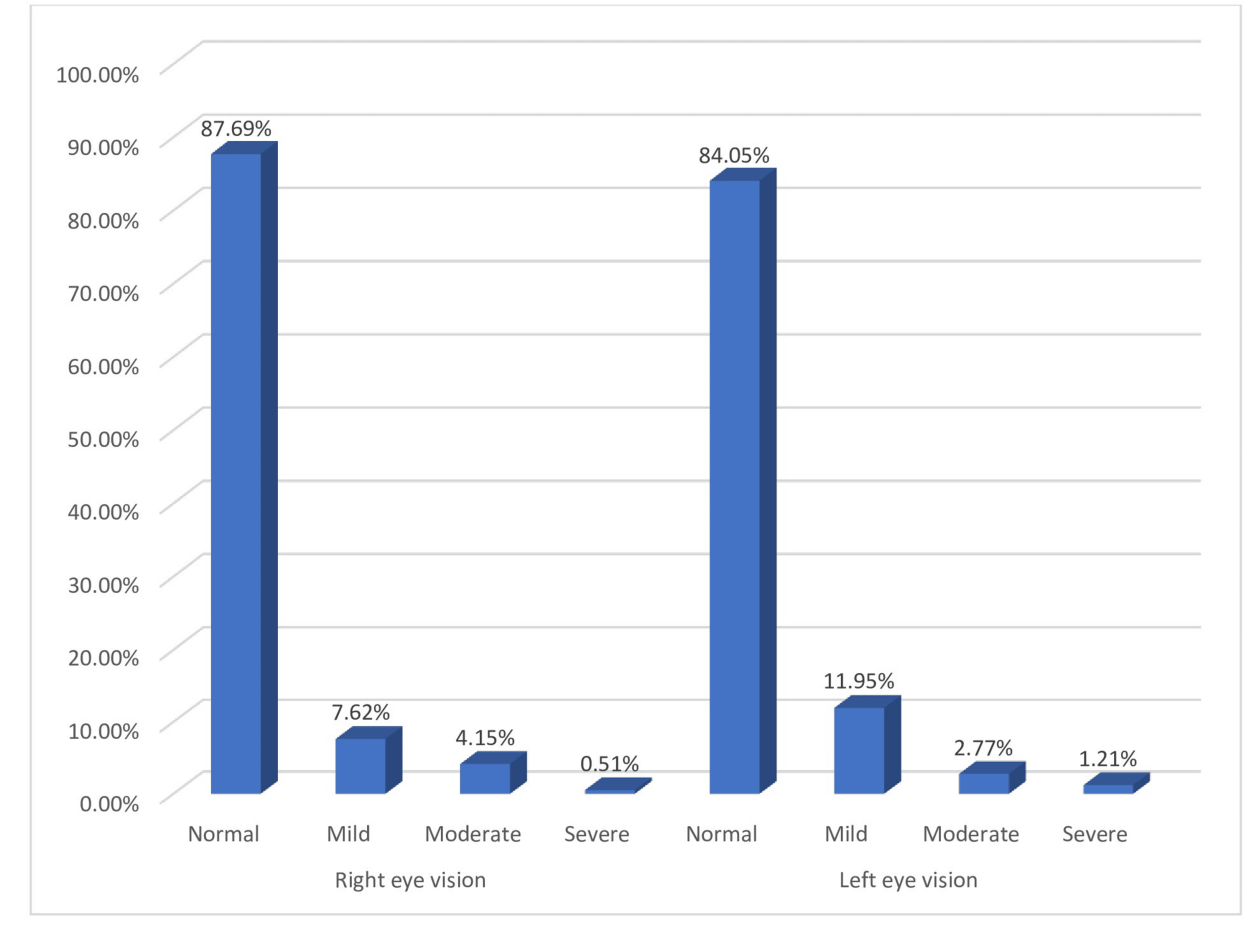
Visual impairment among study participants.

The majority of participants had normal vision in the left eye (485, 84.05%), followed by mild vision impairment (69, 11.95%), moderate vision impairment (16, 2.77%), and severe vision impairment (7, 1.21%). Figure 1 shows the visual impairment among the study participants.

Table 3 shows the association between socio-demographic variables and visual impairment. Age did not show any significant association with visual impairment (p = 0.780). However, a higher proportion of females (16.2%) was found to have visual impairment. Similarly, students residing in urban areas demonstrated a greater prevalence (23.1%). In addition, those with more than two hours of daily screen time had a higher risk of visual impairment. Thus, gender, place of residence, and screen time were found to have a statistically significant association with visual impairment (p < 0.001).

**Table 3:** Association between socio-demographic variables with respect to visual impairment.

Variables	Normal	Visual impairment	Total	P-value
Age				
<20 years	119 (86.2%)	19 (13.8%)	138 (100%)	P = 0.780
21-25 years	249 (82.5%)	53 (17.5%)	302 (100%)	
26-30 years	105 (84.7%)	19 (15.3%)	124 (100%)	
>30 years	12 (92.3%)	1 (7.69%)	13 (100%)	
Gender				
Male	75 (82.5%)	13 (14.8%)	88 (100%)	P < 0.001
Female	410 (83.8%)	79 (16.2%)	489 (100%)	
Residence				
Rural	295 (89.4%)	35 (10.6%)	330 (100%)	P < 0.001
Urban	190 (76.9%)	57 (23.1%)	247 (100%)	
Screen time				
1-2 hours	139 (76.4%)	43 (23.6%)	182 (100%)	P < 0.001
>2 hours	346 (87.6%)	49 (12.4%)	395 (100%)	
Total	485 (84.1%)	92 (15.9%)	577 (100%)	

## Discussion

Refractive errors are one of the most prevalent but frequently overlooked causes of visual impairment (VI), even among young adults like health professional students. While the adolescent age group is commonly thought of as being less risky, our experience shows that VI is by no means uncommon in nursing students. With the aid of the WHOeyes app, a field-tested and transportable visual acuity measuring digital device, a significant percentage of students were found to have impairment of varying severity, even though the majority were between 20 and 22 years old. Timely detection in such an age group is paramount, as uncorrected refractive disorders can impact academic performance, clinical training, and quality of life.

In the present study, VI was found in 12.28% of right eyes and 15.9% of left eyes. Right eye vision test showed that the majority of participants had normal vision (506, 87.69%), followed by mild vision impairment (44, 7.62%), moderate vision impairment (24, 4.15%), and severe vision impairment (3, 0.51%). Left eye vision test showed the majority of them had normal vision (485, 84.05%), mild vision impairment (69, 11.95%), moderate vision impairment (16, 2.77%), and severe vision impairment (7, 1.21%), respectively. While most of them had normal vision, these numbers are clinically noteworthy given the young age of the population. Such trends were reported by Ramesh Masthi et al. [6], who identified that the majority of young adults had normal vision, with fewer than 10% showing mild or moderate impairment. They also reported no significant correlation between VI and screen usage or lifestyle, whereas the present study showed a significant association between screen time and VI.

Similar findings have also been described in neighboring countries. Shrestha et al. [7] found a 14.2% prevalence of uncorrected refractive errors among Nepalese medical and nursing students, with females having a higher prevalence of myopia. Likewise, in our population, with 84.7% females, 16.2% had VI.

Uncorrected refractive errors globally are the most common cause of avoidable VI and blindness. The Global Burden of Disease Study 2019 determined that refractive errors were the leading cause of moderate and severe VI and disproportionately affected low- and middle-income countries [8]. In Southeast Asia, the increasing prevalence of refractive error among young adults and adolescents has been attributed to higher screen exposure, urbanization, and less outdoor activity [9,10]. With close to 12-16% of our participants showing impairment, our results add strength to the evidence for this increasing public health issue.

A particular strength of our research was the application of the WHOeyes. Validated on the ETDRS (Early Treatment Diabetic Retinopathy Study) chart with very good accuracy [11], its portability and automated nature make it especially suitable for large-scale and low-resource environments. This is significant for our study, where 58.4% of the participants were from rural areas. Prior research [12,13] has also established the practicality of smartphone vision testing in low-resource settings as a basis for wider use of such technology in community screening schemes.

In the present study, age was not a significant factor with VI (p = 0.780), presumably because the range of participants was so restricted. But gender, residence, and screen use were all strongly correlated (p < 0.001). Females had a higher prevalence (16.2%), urban dwellers reported more impairment (23.1%), and subjects with more than two hours of screen exposure per day were more likely to be impacted. These correlations indicate lifestyle and environmental influences on the development of refractive error.

Interestingly, the results for screen exposure are different from those of Ramesh Masthi et al. [6], who found no such correlation. Nevertheless, other research has implicated long-term screen exposure with digital eye strain and accommodative stress [14]. A review by Sheppard and Wolffsohn [14] also corroborates the involvement of near activities and prolonged accommodation in visual fatigue, which aligns with our findings. Variation in methodology and population demographics can explain differences between studies, but our findings reinforce the growing concern over digital device use on student eye health.

From a public health standpoint, these results emphasize the need for regular vision screening in schools. Target interventions for high-risk populations like young women, urban dwellers, and heavy screen users are necessary. In addition, making corrective lenses available at a reasonable cost, especially to students from lower socio-economic groups, continues to be a policy priority.

There are a few limitations in this study. First, because data were gathered at a particular point in time, its cross-sectional design makes it difficult to determine causal relationships between socio-demographic factors and visual impairment. Second, the results may not be applicable to other students or the larger young adult population because the sample was limited to nursing students at a single institution.

## Conclusions

In conclusion, this research provides evidence that most young nursing students possess normal vision, yet an important proportion have mild to moderate VI owing mainly to uncorrected refractive defects. This emphasizes the demand for routine vision screening procedures, especially with uncomplicated and validated digital tools such as WHOeyes. Correcting refractive errors at an early age is important in enhancing academic functioning, quality of life, and long-term productivity of future health professionals.

Recommendations include implementing routine vision screening in educational institutions, which is one of the main measures to combat the growing burden of visual impairment and refractive errors among young adults. This will allow for early detection and repair. Awareness campaigns should emphasize the value of routine eye exams, preventive actions, and education about eye health. To lessen digital eye strain, students need to be instructed about safe screen habits and good ergonomics rules. Accessibility in remote locations is ensured by using digital screening technologies such as WHOeyes. Furthermore, promoting outdoor activities encourages lifestyle modifications that lessen refractive problems associated with prolonged near employment.
